# Factors influencing mother’s participation in *Posyandu* for improving nutritional status of children under-five in Aceh Utara district, Aceh province, Indonesia

**DOI:** 10.1186/s12889-016-2732-7

**Published:** 2016-01-22

**Authors:** Cut Nazri, Chiho Yamazaki, Satomi Kameo, Dewi M.D. Herawati, Nanan Sekarwana, Ardini Raksanagara, Hiroshi Koyama

**Affiliations:** 1 Department of Public Health, Gunma University Graduate School of Medicine, Maebashi, Japan; 2 Faculty of Medicine, Universitas Padjajaran, Jatinangor, Indonesia

## Abstract

**Background:**

*Posyandu*, or *pos pelayanan terpadu* (integrated service post), is a community-based activity for health services in Indonesia. According to the Indonesian Basic Health Survey, the prevalence of children under five in Indonesia who suffered from being underweight was 19.6 %. The wasting was 12.1 % and the stunting was 37.2 % in 2013, and these values have not changed greatly from 2007; much greater than the WHO targets of, less than 10 % underweight, 5 % wasting, and 20 % stunting. In Aceh were 26.6, 16.8, and 43.3 %, respectively. Also, the participation percentages of mothers to *Posyandu* was about 45 %, far below the national target of 100 %. In Aceh Province, the percentage was even lower (34 % in 2013). This study aimed to investigate the factors influencing participation of mothers in *Posyandu*.

**Methods:**

This research used a cross-sectional design with sample of mothers who had children under five. They were chosen by multistage random sampling. Sample size was determined by the WHO formula. Face-to-face interviews were carried out using a questionnaire. The questionnaire consisted of items about socio-demographic characteristics, satisfaction with *Posyandu* services, attitude towards *Posyandu* benefits, and intention to attend *Posyandu*. The collected data were analyzed by using EZR (version 1.21). Fisher’s exact test was performed to examine the associations between the socio-demographic factors, attitude, satisfaction, and intention covariates with participation. Logistic regression was used to describe the strength of the relationship between the predictor variables and participation.

**Results:**

There were no significant differences in age, marital status, education level, occupation, family size, and distance to *Posyandu* between low participation group except for the monthly household income. Among the socio-demographic factors, only monthly household income had a significant association with the frequency of mothers’ participation. Satisfaction, attitude, and intention were associated with participation. The logistic regression showed that monitoring the nutritional status of children under five was the main reason that mothers participated in *Posyandu*. Mothers who were satisfied with the *Posyandu* services were more likely to attend than those who were dissatisfied. Respondents with intention to participate in *Posyandu* every month were more likely to attend than those who did not intend to attend every month. Households with low income were more likely to participate in *Posyandu* than households with high income.

**Conclusion:**

Household income, mothers’ satisfaction with *Posyandu* services, attitude towards *Posyandu* benefits and intention to attend *Posyandu* affect the participation frequency of the mother. In addition, monitoring the nutritional status of children under five was the main reason respondents attend *Posyandu*. Improving the quality of *Posyandu* services and providing qualified resources are needed to promote mothers’ participation.

## Background

Under-nutrition is the outcome of insufficient food intake and repeated infectious diseases. It is a major public health problem that needs more attention because it leads to the loss of a generation. This contributes to the deaths of approximately 5.6 million children under five in the developing world each year. It triggers poor school performance and dropout, decreased productivity, threatens the ability of girls to bear healthy children in the future and perpetuates a vicious cycle [1].

According to the Indonesian Basic Health Survey, the prevalence of children under five in Indonesia who suffered from being underweight (weight for age is below -2 standard deviations of the WHO Child Growth Standard median) was 19.6 %. The wasting (weight for height is under -2 standard deviations of the WHO Child Growth Standard median) was 12.1 % and the stunting (height for age is below -2 standard deviations of the WHO Child Growth Standard median) was 37.2 % in 2013, and these values have not changed greatly from 2007 [2, 3]; much greater than the WHO targets of, less than 10 % underweight, 5 % wasting, and 20 % stunting [2–4]. In Aceh were 26.6, 16.8, and 43.3 %, respectively [2–4].

In 1975, the Indonesian government established the Development of Village Community Health (*PKMD, Pembangungan Kesehatan Masyarakat Desa*). PKMD is a health development strategy that applies the principles of mutual work and non-governmental organizations. It preceded an international agreement on a similar concept; known as the Primary Health Care (PHC), as stated in the Alma Ata Declaration in 1978. Nevertheless, PKMD activities were compartmentalized [5], nutrition improvement, diarrhea prevention, immunization and family planning were managed by different sectors. These compartmentalization caused difficulties in coordinating programs, resource inefficiencies, and were a disadvantage of *PKMD* [5]. To address these problems, in 1984, the Indonesian government merged these activities into an integrated service program called *Posyandu* (*pos pelayanan terpadu*).

*Posyandu* (Health and Nutrition Integrated Service Center) aim to provide basic health services such as family planning, mother and child health, nutrition (growth monitoring, supplemental feeding, vitamin and mineral supplementation and nutrition education), immunization, and disease control (diarrhea prevention) [6]. *Posyandu* is supported by Ministry of Health (act as the leading sector), Family Planning Board, and Ministry of Internal Affairs. However, considering *Posyandu* is a community-based effort, it has to be carried out by, from, and for the community, thus community participation is essentials to implement the basic health effort. In order to function, *Posyandu* need the village health post volunteers called Cadres. They are voluntarily selected from community members to organize the acitivies of *Posyandu. Puskesmas* (Primary Health center; as the sub-district representative of Ministry of health) will train the cadres until they are able to provide basic health care services required. The medical doctor or midwife from Puskesmas also has to help cadre held every activities, as written in law about *Puskesmas* (Indonesian Health Ministry Regulation No. 75 Year 2014). Community leader also has to support by conducting a fund raising and encourage the community to actively participate in the implementation of *Posyandu*. The community is encouraged to run the *Posyandu* once a month in every village.

Levels of *Posyandu* are divided into 4 levels. First level is *Posyandu Pratama*, this *Posyandu* has limited cadres (less than 5 cadres) and the activities are not conducted monthly. Second level is *Posyandu Madya* which has carried out the activities more than 8 times per year and have 5 cadres or more. The coverage of 5 activities is still low, under 50 %. Third level is *Posyandu Purnama*, its’ criteria are same with *Posyandu Madya*. However, it has independent fund managed by community itself. The participants involving in are less than 50 %. *Posyandu mandiri* is the last level. It has as same criteria as *Posyandu Purnama*, yet it has 50 % or more of participants [7].

**Fig. 1:**
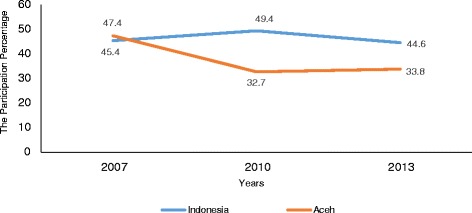
The percentage of participation in *Posyandu*. Source: Indonesian basic health survey 2007, 2010, and 2013

Community participation is the active involvement of people from communities in health activities [8]. In Indonesia, the participation percentage of mothers to *Posyandu* was about 45 % [2–4], far below the national target of 100 %. In Aceh Province, the percentage was even lower (34 % in 2013) (Fig. 1) [4]. Therefore this study aimed to investigate the factors influencing mother’s participation in *Posyandu* for improving the nutritional status of children under five in the Aceh Utara District of Aceh Province.

## Methods

### Subjects of research

This research used a cross-sectional design with sample of mothers who had children under five. The participants were selected randomly through multistage sampling. First stage we selected sub-districts, in each sub-district we chose villages, then the participants were chosen randomly from household list provided by the village government and *Puskesmas* using a random number table. All of *Posyandu* in Aceh Utara are *Posyandu Madya* category. The inclusion criteria were individuals who had an identity card (*KTP, kartu tanda penduduk*) and agreed to participate to this study. Those having visual and hearing impairment, or who are unable to communicate due to intellectual or mental disorder were excluded. A sample size of 384 mothers with children under five years was calculated to be required based on the sample size determinants in health studies by the World Health Organization (WHO) [9]. Informed consent was given when the surveyors visited the subjects. Face-to-face personal interviews using a questionnaire were conducted to those who agreed to participate.

### Instrument

The questionnaire consisted of items assessing socio-demographic factors, mothers’ satisfaction with *Posyandu* services, attitudes toward *Posyandu* benefits, intention to attend *Posyandu*, participation frequency, and reasons for attending *Posyandu*. The socio-demographic items contained the date of birth, marital status, the highest level of education completed, occupation, household income (monthly), the number of people living in the household, and the house distance to *Posyandu*. The satisfaction, attitude, intention, participation frequency, and reasons for attending *Posyandu* encompassed in the following questions: -how satisfied mothers were with the *Posyandu* services for children under five, if they agreed that attending *Posyandu* makes children healthy, would mothers attend *Posyandu* for monitoring the child’s nutritional status, how often has the mother attended *Posyandu* to weigh the child, and what is the reason for attending *Posyandu*.

### Data collection

The research was conducted in March and April in 2014. Data was collected by face-to-face interview using a questionnaire. The surveyor administered questionnaire by reading out each question and record respondent’s response. The surveyor were the nutrition staffs of *Puskesmas* who were acquainted by the community members and experienced in recording *Posyandu* data monthly. The study instrument was written in Indonesian language, but the interview was performed using Indonesian language and common Acehnese (local dialogue).

### Data analysis

The data were analyzed using statistical software (EZR version 1.21) and all of the statistical tests used a significant level of *p* < 0.05 and 95 % confidence intervals [10].

The socio-demographics, mothers’ satisfaction, attitude, intention, and participation were described in contingency tables (Table 1) and analyzed by Fisher’s exact tests. The logistic regression analysis was performed to describe the strength of the relationship between the predictor variables and participation. The predictor variables were the household income, mothers’ satisfaction with *Posyandu* services, attitude towards *Posyandu* benefits, intention to attend *Posyandu*, and reasons of respondents for participating in *Posyandu*. To determine which set of predictor variables is the best fit for multivariate analysis, *Akaike* Information Criterion (AIC) was performed [11].

**Table 1:** The category and coding of variables

Variables	Group	Coding
Age category	≤30 years	0
	>30 years	1
Marital status	Divorced and widow	0
	Married	1
Education level	Basic education	0
	Higher education	1
Occupation	Unemployed	0
	Private sector and entrepreneur	1
Household income (monthly)	≤1 million IDR (≤90.9 USD)	0
	>1 million IDR (>90.9 USD)	1
Family size	≤5 persons	0
	>5 persons	1
Distance to Posyandu	≤3 km	0
	>3 km	1

### Ethical considerations

This study was reviewed and approved by the Epidemiologic Research Ethics Committee of Gunma University Faculty of Medicine and Health Research Ethics Committee of Universitas Padjadjaran. Permission to conduct this study in the area was obtained from the district government and the Health Office of Aceh Utara District. The study objectives and rights of participants were explained and verbal informed consent was obtained from all participants before collecting data.

## Results

### Demographic characteristics

We recruited 388 mothers to fulfill the required minimum sample size with the possibility of drop out. No one declined participation, however, three subjects were excluded due to the incompleteness of the questionnaire. Thus, 385 mothers took part and completely answered the questions.

The characteristics of the respondents are indicated in Table 2. There were no significant differences in age, marital status, education level, occupation, family size, and distance to *Posyandu* between low (≤6 time/year) and high (>6times/year) participation group except for the monthly household income (*p* = 0.047).

**Table 2:** Association of socio-demographic factors with participation frequency among the mothers

Variable	Participation		*N* = 385 (%)	*P* value
	≤6 times a year	>6 times a year		
	*N* = 192 (%)	*N* = 193 (%)		
Age category				
≤30 years	105 (50)	105 (50)	210 (54.5)	1
>30 years	87 (49.7)	88 (50.3)	175 (45.5)	
Marital status				
Divorced and widow	7 (33.3)	14 (66.7)	21 (5.5)	0.177
Married	185 (50.8)	179 (49.2)	364 (94.5)	
Education Level				
Basic education	38 (50.7)	37 (49.3)	75 (19.5)	0.898
Higher education	154 (49.7)	156 (50.3)	310 (80.5)	
Occupation				
Unemployed	124 (48.2)	133 (51.8)	257 (66.8)	0.388
Private sector and entrepreneur	68 (53.1)	60 (46.9)	128 (33.2)	
Household Income^a^(monthly)				
≤1 million IDR	156 (47.7)	171 (52.3)	327 (84.9)	0.047
>1 million IDR	36 (62.1)	22 (37.9)	58 (15.1)	
Family size				
≤5 persons	145 (48.2)	156 (51.8)	301 (78.2)	0.219
>5 persons	47 (56.0)	37 (44.0)	84 (21.8)	
Distance to *Posyandu*				
≤3 km	74 (54.4)	62 (45.6)	136 (35.3)	0.202
>3 km	118 (47.4)	131 (52.6)	249 (64.7)	

Fisher’s exact test^. a^1 million IDR = 90.9 USD (10^th^ of March 2014 exchange rate)

### Association of socio-demographic factors with participation frequency

Since the *Posyandu* takes place once a month, we divided the subjects who participate less than or more than half of the *Posyandu* in a year, and analyzed the socio-demographic factors with participation frequency. Among the socio-demographic factors, only monthly household income had a significant association with the frequency of mothers’ participation (*p* < 0.05); mothers of household with >1 million IDR participate less frequent to the *Posyandu* (Table 2).

### Association of the mothers’ satisfaction, attitude, and intention with participation frequency

Although the majority of mothers were satisfied with *Posyandu* services (69.9 %) and had positive attitude towards *Posyandu* benefits (71.4 %), half of respondents had no intention of participating in *Posyandu* every month (50.1 %). Satisfaction, attitude, and intention were associated with participation (*p* < 0.001) as shown Table 3; mothers who are satisfied, have positive attitude and have intention to participate every month participate more frequent to *Posyandu*.

**Table 3:** Association of the mothers’ satisfaction, attitude, and intention with participation frequency

Variable	Participation		*N* = 385 (%)	*P* value
	≤6 times a year	>6 times a year		
	*N* = 192	*N* = 193		
Satisfaction				
Dissatisfied	109 (94.0)	7 (6.0)	116(30.1)	<0.001
Satisfied	83 (30.9)	186 (69.1)	269 (69.9)	
Attitude				
Negative	83 (75.5)	27 (24.5)	110 (28.6)	<0.001
Positive	109 (39.6)	166 (60.4)	275 (71.4)	
Intention				
Not every month	147 (76.2)	46 (23.8)	193 (50.1)	<0.001
Every month	45 (23.4)	147 (76.6)	192 (49.9)	

Fisher’s exact test

### Predictor variables of participation (multivariate analysis)

The multivariate analysis showed that mothers whose reason were growth monitoring were more frequent participants in *Posyandu* (odds ratio: 16.30; 95 % CI = 2.98–89.10) than those attending immunization reasons (Table 4). Respondents satisfied with *Posyandu* services were more likely to attend *Posyandu* (odds ratio: 13.70; 95 % CI = 5.42–31.00) than those who were dissatisfied. Mothers intending to take their child to *Posyandu* every month were more likely to participate in *Posyandu* (odds ratio: 5.89; 95 % CI = 3.28–10.60) than those not intending to take their child every month. Subjects with low family incomes were more likely to attend *Posyandu* than subjects with high incomes (odds ratio: 0.4; 95 % CI: 0.18–0.94). The AIC value of this model was 306.63. We excluded attitude because the AIC value was the same with or without it. Then, we predicted that there is a correlation between the covariates of attitude and intention and, attitude and satisfaction.

**Table 4:** Predictor variables of participation (multivariate analysis)

Variable	Odds Ratio	95 % Confident Interval	AIC
Satisfaction	13.70	5.42–31.40	306.63
Intention	5.89	3.28–10.60	
Household income	0.41	0.18–0.94	
Reason:			
- Immunization	Reference		
- Monitoring nutritional status of children under five	16.30	2.98–89.10	
- Near to *Posyandu*	4.07	0.74–22.40	
- Free or no charge	2.98	0.51–17.30	
- Supplementary feeding	0.49	0.03–7.14	

*AIC Akaike* information criterion

For proofing this correlation, we examined these covariates with bivariate analysis.

### Association of satisfaction with intention and attitude and, attitude with intention

Table 5 show that there was a significant association between satisfaction and intention and, attitude and intention (*p* < 0.001). Table 6 also presented the significant association between satisfaction and attitude (*p* < 0.001). Satisfied respondents and those with positive attitude intended to attend *Posyandu* every month. Satisfied subjects had positive attitude towards the benefits of *Posyandu*.

**Table 5:** Association of satisfaction and attitude with intention

Variable	Intention		*N* = 385	*P* value
	Not every month	Every month		
	*N* = 193	*N* = 192		
Satisfaction				
Dissatisfied	95	21	116	<0.001
Satisfied	98	171	269	
Attitude				
Negative	78	32	110	<0.001
Positive	115	160	275	

Fisher’s exact test

**Table 6:** Association of satisfaction with attitude

Variable	Attitude		*N* = 385	*P* value
	Negative	Positive		
	*N* = 110	*N* = 275		
Satisfaction				
Dissatisfied	72	44	116	<0.001
Satisfied	38	231	269	

Fisher’s exact test

### Reason for participation

**Fig. 2:**
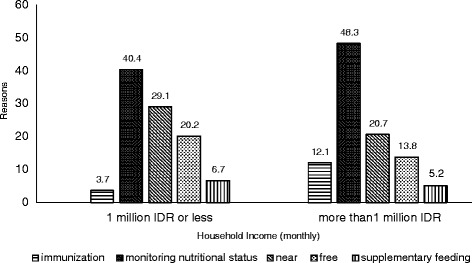
Reasons for participation in *Posyandu* categorized by household income. The main reason of both groups was monitoring nutritional status in attending *Posyandu*

**Fig. 3:**
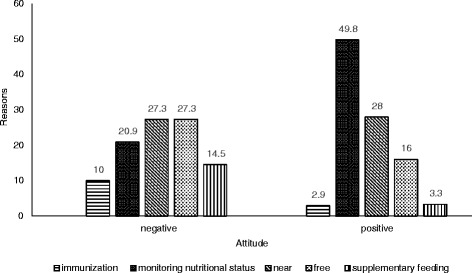
Reasons for participation in *Posyandu* categorized by attitude. The main reasons in negative attitude group were near from *Posyandu* and free/no charge in attending *Posyandu*

**Fig. 4:**
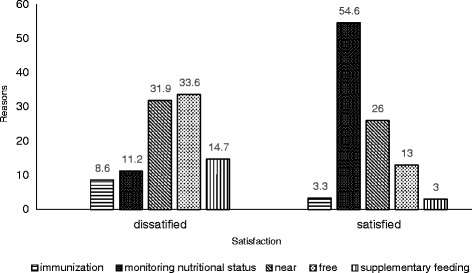
Reasons for participation in *Posyandu* categorized by satisfaction. The main reason for dissatisfied group was free/no charge in attending *Posyandu*

**Fig. 5:**
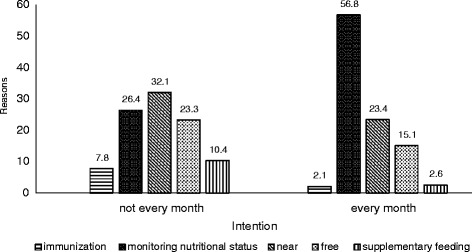
Reasons for participation in *Posyandu *categorized by intention. The main reason for intention not every month group was near from *Posyandu*

The most common reasons respondents participated in *Posyandu* was growth monitoring of their children (Figs. 2, 3, 4 and 5). When categorized by household income, most frequent answer was ‘growth monitoring’ by far, followed by ‘near from my house’ and ‘free’ for both ≤1 million IDR and >1 million IDR groups. The distribution of the reasons for participation showed similar tendency when categorized by attitude, satisfaction, and intention. Approximately 50–55 % of the mothers who have favorable impression towards *Posyandu* (positive attitude, satisfied, intend to attend every month) answered ‘growth monitoring’ as the reason for attending *Posyandu*. Those with unfavorable impressions (negative attitude, dissatisfied, not intend to attend every month) mentioned ‘near’ and ‘free’ as the main reasons for attending *Posyandu*.

## Discussion

We found that the household income had a significant association with the mothers’ participation frequencies, and mothers of higher household income groups showed lower participation. The mothers of high household income is more likely to be working outside home rather than being a housewives, therefore more difficult to manage time to attend *Posyand*u every month [12]. Also, they are financially capable of bringing their children to other health facilities such as *Puskesmas*, general practitioneurs, or midwives to receive the same services at their convenient. In the study on effectiveness of Vitamin A capsule distribution program in Indonesia, one of the services provided by *Posyandu*, mothers gave ‘usually take child to another health facility’ (13.8 %) and ‘too busy’ (2.9 %) as reasons not taking children to the *Posyandu* [13]. However, considering 85 % of respondents of this study were in household income ≤1 million IDR group, the activities provided at free of charge in *Posyandu* is of great significance as all the children can receive much benefit.

This study showed that mothers with positive attitude towards *Posyandu*, satisfied with *Posyandu* service, and/or had intention to attend showed high attendance. In addition, these variables were associated with each other. The results of multivariate analysis showed that satisfaction affect more on the participation of the mothers than intention or attitude. This indicates that interventions that would increase mothers’ satisfaction would promote their attendance to *Posyandu*.

Improving the quality of services is one way and probably the most significant way to increase mother’s satisfaction on *Posyandu* services. Even though *Posyandu* is conducted and nutritional advice is given to the mothers every month, prevalence of malnutrition children remain high in Indonesia and even higher in Aceh. This clearly indicates that current approach is ineffective. In spite of worldwide use of growth monitoring especially in the developing countries, many reports point out that growth monitoring alone would not reduce children’s growth faltering, and ‘good nutrition counselling is paramount for growth promotion and is often done badly’ [14]. *Posyandu* in is unexceptional. Training of cadres is too brief and not practice-oriented, result in cadres have neither the knowledge nor skills to communicate effectively with caregivers [14]. Even cadres themselves feel that counseling is necessary to be improved [15]. Advices provided by cadres are often inappropriate or superficial. For example, mothers of a child with low body weight are advised to give more food to the child, but it is impractical for the poor family. It is clear that adequate and periodic training of cadres is definitely needed so that all the cadres meet certain standards, especially they are volunteers with various levels of educational background. But it is not as easy in practice since the *Posyandu* activities are carried out by the community, and the community must secured the financial resources for cadres’ training.

The leading reason for the mothers to attend *Posyandu* was children’s growth monitoring, far more frequent (41.6 %) than other reasons. Yet prevalence of underweight, wasting, stunning of children under 5 in Ache is higher than Indonesia’s national level, indicating suitable care is not provided to the children at risk of malnutrition. To reduce children with under-nutrition or growth faltering, such children must be identified first without oversight, then specific counseling and efficacious advices should be given by health professionals, and special support to high-risk children such as extra follow-up, food, frequent counseling sessions, and weekly weighing should be provided. The appropriate allocation of roles between cadres and health professionals in this process may be necessary for more effective intervention. Cadres should be trained to accurately take measurements and plot on the chart and judge whether the child is maintaining good growth. In case they detect growth faltering, cadres should refer the mothers to a midwife who is also present at *Posyandu* for special feeding and nutrition advices. Improvement of child’s nutritional status by detecting children at risk and providing appropriate advices by health professionals would enhance the quality of services, therefore increasing mother’s satisfaction.

Besides capacity building of cadres, education of mothers is also important. Growth charts can make children’s growth visible to caregivers and it can be used as educational tool, but mothers must have ability to understand the growth curve. Understand what growth curve is denoting would promote mothers’ interests in their children’s nutrition and the way they care their children. In the implementation of community-based growth monitoring project in South Africa, caregivers were pleased that they understood the link between appropriate weight gain, nutrition and their children’s health, and gained sense of empowerment through better understanding of what made their children health and how to check this [16]. Prior to the project, only 3 % of infants were taken to the clinic specifically for growth monitoring; the mothers were not motivated because the rationale was never explained to them. Studies in Indonesia showed lack of caregiver’s knowledge about the benefit of vitamin A is associated with the low rates of participation in the national vitamin A capsule program [17, 18]. These studies indicate that advancement in mothers’ knowledge and understanding would increase their interest in the nutritional conditions of their children and would motivate their attendance to community health programs such as *Posyandu*.

The health care system in Indonesia emphasizes community empowerment, and organization of *Posyandu* activities are responsible of village community. Therefore, the support from heads of the village and village health committees are essential for the success of *Posyandu* activities. Research on close-to-community (CTC) services in Indonesia reported the villages performing well (in terms of utilization of services) had much more supportive and active village heads and other stakeholders than those performing less well [19]. The village heads, community leaders, and religious leaders are influential in the community and useful in raising the community’s awareness of the importance of *Posyandu* activities. Community leaders and health sector collaborators should disseminate information through media campaigns and village meetings. In addition, they have authority to allocate funding to provide appropriate facilities and instruments for *Posyandu*. Holding leadership workshops for the community leader for good governance or stewardship may be another possible intervention to promote active participation of the community, and in turn child health promotion of the area.

About one third of the mothers had unfavorable impression towards *Posyandu*, and their reasons for attending *Posyandu* were ‘closeness to home’ and ‘free of charge’ of the *Posyandu* services. Many studies reported the distance to the health facilities are the barriers to accessing health services [20–22]. Our findings showed that closeness in distance prompt the attendance to *Posyandu*; the mothers who are not satisfied and not motivated would attend *Posyandu* if it is close to home. Holding *Posyandu* at the village-level is indispensable in child health because being readily accessible would increase chance for the undernourished children to be detected, and would be opportunities to provide information or advice to mothers. Indonesia has already established community-based health care delivery system (*Posyandu*) that is remain an issue to many developing countries. Increasing quality of services of *Posyandu* is the challenges for the future. Improvement of coordination and referral system of health providers (midwives, cadres, and *Puskesmas* staffs), implementation of formal and regular training of cadres are potential areas for remediation.

The limitation of this study is that the study sample may not represent the population of mothers of children under five living in the Aceh Utara District. Additionally, a deeper exploration of the influencing factors of participation through the combination of quantitative and qualitative studies is needed.

## Conclusion

In our study, household income, mothers’ attitude towards the benefits of *Posyandu*, satisfaction with Posyandu services, and intention to attend *Posyandu* significantly affected participation frequency of the mothers. In addition, growth monitoring of children under five was the reason respondents attended *Posyandu*. Hence, improving the quality of *Posyandu* services and providing qualified resources can promote the participation of mothers.
